# Altered dynamics of mitochondria and reactive oxygen species in the erythrocytes of migrating red-headed buntings

**DOI:** 10.3389/fphys.2023.1111490

**Published:** 2023-02-09

**Authors:** Nitin Bhardwaj, Anit Kumar, Neelu Jain Gupta

**Affiliations:** ^1^ Department of Zoology and Environmental Science, Gurukula Kangri (Deemed to be University), Haridwar, Uttarakhand, India; ^2^ Department of Zoology, Chaudhary Charan Singh University, Meerut, Uttar Pradesh, India

**Keywords:** erythrocytes, apoptosis, reactive oxygen species, mitochondrial potential, migration, photoperiod

## Abstract

**Background:** Blood antioxidants provide propensity to mitigate reactive oxygen species (ROS) apart from other oxidative challenges during a high-energy state of migration in night migratory songbirds. The study investigated the modulation of erythrocytes, mitochondrial abundance, hematocrit changes, and relative expression of fat transport-related genes during migration in red-headed buntings (*Emberiza bruniceps*). We hypothesized an increase in antioxidants along with the mitigation of mitochondria-related reactive oxygen species elevation and consequential apoptosis occurring during migration.

**Methods:** Male red-headed buntings (*n* = 6) were placed under short days (8 h of light and 16 h of dark, 8L:16D)/long days (14L:10D) and photo induced to simulated non-migratory, nMig; pre-migratory, pMig; and migratory, Mig, states. Erythrocyte shape, reactive oxygen species production, mitochondrial membrane potential (MMP), reticulocyte proportion, and apoptosis were analyzed using flow cytometry and relative expression of fat metabolizing and antioxidant genes was measured by using qPCR.

**Results:** There was a significant increase in hematocrit, erythrocyte area, and mitochondrial membrane potential. Reactive oxygen species and apoptotic erythrocyte proportion declined in the Mig state. The changes in antioxidant genes (SOD1 and NOS2), fatty acid translocase (CD36), and metabolic (FABP3, DGAT2, GOT2, and ATGL) genes showed a significant increment during the Mig state.

**Conclusion:** These results suggested that adaptive changes occur in mitochondrial behavior and apoptosis of erythrocytes. The transition in erythrocytes, antioxidant genes, and fatty acid metabolism gene expressions suggested differences in regulatory strategies at the cellular/transcriptional level during different states of simulated migration in birds.

## Introduction

Physiological revamping in night migratory songbirds is associated with migratory success. These animals fly long distances every autumn and spring, from breeding to wintering grounds and contrariwise. To sustain intense migration exercise, flight muscles must receive unhampered oxygen supply, putting the blood vessels into migratory hyperemia. Birds’ body physiology redirects to the series of tissue-dependent adaptive changes (Handby et al., 2022). Heart enlargement (Trivedi et al., 2014), hematocrit increase (Butler, 2016), the liver hypofunction aiding fat mobilization from adipose tissue (Guglielmo, 2010), altered muscle size dynamics with insulin-like growth factor, and IGF1 rise (Price et al., 2011) are prominent physiological changes. Blood mediates the multitude of supplies such as oxygen and energy metabolites amid heightened hypoxia-reoxygenation physiology (McWilliams et al., 2021).

In blood, erythrocytes are hemoglobin (Hb)-containing cells that exhibit dynamic morphology to aid seasonal physiological changes in birds (Bańbura et al., 2007). The total Hb concentration in erythrocytes is proportional to the oxygen binding capacity. Many avian species exhibit changes in Hb concentration with seasonal and other physiological changes, such as moult, and might relate to birds’ ability to fulfill their respective oxygen requirements (Kostelecka-Myrcha, 1997; Minias, 2015). The hematocrit (packed cell volume, PCV) indicates erythrocytes’ proportion in blood volume, which may (Butler, 2010) or may not change during avian migration (Fair et al., 2007). Smaller and more elongated erythrocytes during migration facilitated oxygenation/deoxygenation and aerobic metabolism (Soulsbury et al., 2021). Refueling compensated hematocrit lowering in late-arriving bar-tailed godwits, thus correcting in-flight light anemia (Merila and Svenson, 1995; Piersma et al., 1996; Jenni et al., 2006). In zebra finch, erythrocytes get affected due to the enhanced reactive oxygen species (ROS) levels (Stier et al., 2013). In migratory birds, no reports on erythrocyte dynamics and their putative role on alleviating exercise accrued ROS are available.

ROS are short-lived by-products (Halliwell, 2011; Winterbourn, 2015) of high mitochondrial activity, a prerequisite of elevated metabolism (Dmitry et al., 2014). Inside cells, ROS levels above homeostatic balance aggravate apoptosis (Kamata et al., 2005), a well-executed suicidal plan of the cell, induced by high mitochondrial activity. In addition to cellular ROS, nitro-oxidative damage is also indicated by NOS2 (nitric oxide synthase) levels (Bredt and Snyder, 1994). Antioxidants such as superoxide dismutase (SOD1) show the first line of cellular defense to counteract ROS and other oxidants. In mitochondria, ubiquitin-mediated degradation *via* proteolysis regulates energy metabolism (Lavie et al., 2018).

Regular flight during migration stimulates antioxidant protection in addition to fat catabolic pathways. For example, mitochondrial fatty acid oxidation co-occurs with a rise in palmitoylethanolamide, PEA levels, which have anti-inflammatory and cannabinomimetic properties (Gupta et al., 2020). Increased transcriptional activity of genes (fatty acid transporter, CD36; fatty acid binding protein, FABP; glutamic-oxaloacetic transaminase; GOT2, diacylglycerol acyl transferase DGAT2 and adipose triglyceride lipase, ATGL) implicated in fat internalization, transport, and breakdown in the liver and/or muscle has been shown in buntings (Sharma et al., 2021). Furthermore, nucleated avian erythrocytes are capable of exhibiting cellular metabolism (Stier et al., 2013). Relative expression of genes of energy metabolism in avian erythrocytes has never been investigated alongside mitochondrial membrane potential (MMP), ROS, and apoptosis during metabolic stress of migration. It is also possible that birds achieve this feat through increased erythropoiesis.

Migratory buntings (*Emberiza* species) exhibit pre-migratory hyperphagia, an increase in body weight, hormones (for example, thyroxine; please see Jain and Kumar, 1995), trigger of intense night-flight behavior (Gupta and Kumar, 2013), and the metabolic ability to support high aerobic capacity (Gupta et al., 2020). In the present study, we sought to comprehensively outline seasonal changes in blood biochemistry and cytology of red-headed buntings, such as reticulocyte regeneration, hematocrit changes, MMP, ROS, apoptosis, and changes in the concentration of selected genes implicated in the ROS and energy metabolism. The flow cytometry method was used for counting and analyzing size range using optical detection of erythrocytes, enabling inherent analysis of several 10,000 cells within a shorter time in addition to reducing the statistical noise. Specific antibodies included 1) anti-transferrin that binds to surface transferrin receptors of reticulocytes, 2) annexin V for analyzing the apoptotic cells, 3) CM-H2DCFDA stain for ROS, and 4) mitotracker red stains for MMP analysis were used. We hypothesized an increment in antioxidants in response to the ROS elevation, in addition to a change in the mitochondrial functioning during migration. We also predicted an altered ROS and apoptosis, associated elevation in fat metabolizing gene levels, and antioxidant gene in blood, as consequences of migration.

## Materials and methods

### Birds

**FIGURE 1:**
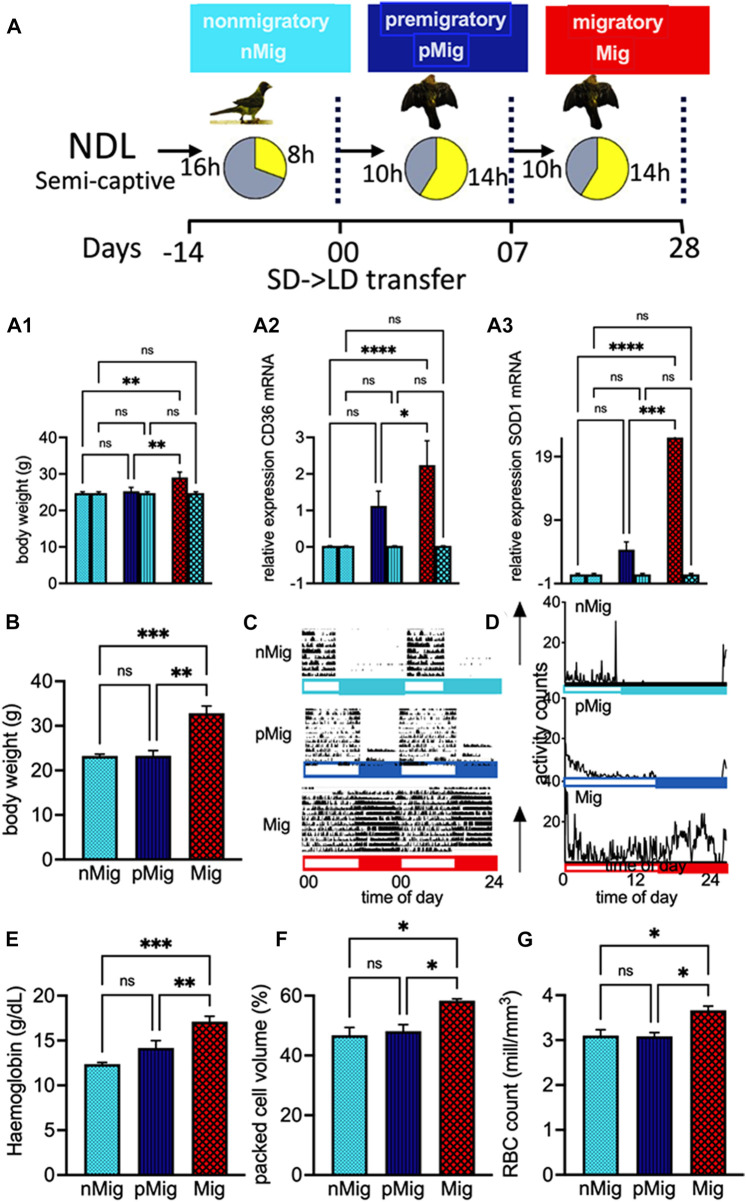
**(A)** Experimental design. Male red-headed buntings, *Emberiza bruniceps*, either in activity cages (*n* = 6) or in two groups (*n* = 6, each) were brought indoors from natural daylength, NDL, conditions during February and placed under a short photoperiod of 8 h of light and 16 h of dark (SD, 8L:16D) for 14 days. Birds in six activity cages and one group were transferred to long days (LD, 14L:10D) on day 0. **(A.1–A.3)** Comparison of body weight (g) and mRNA expression of CD36 and SOD1 of groups 2 and 3, the latter continuously held under SD. Buntings exhibited non-migratory (*nMig*, cyan blue), pre-migratory (*pMig*, blue), and migratory (*Mig*, red) annual life history states, on days 0, 7, and 28 of blood sampling. **(B)** Mean (±SEM) of body weight of *nMig*, *pMig*, and *Mig* buntings. **(C)** Double-plotted actogram representative of *nMig*, *pMig*, and *Mig* buntings. **(D)** Mean activity profile of buntings (*n* = 6), in Figure 1A. Open and closed bars on the X-axis show light and dark phases, respectively. **(E)** Mean (±SEM) of hemoglobin (g/dL), **(F)** mean (±SEM) of packed cell volume (%), and **(G)** mean (±SEM) of red blood cells in blood (millions/millimetre^3^). Data are represented as mean ± SEM. Asterisk (*) over the line on the bar indicates a significant difference between *nMig*, *pMig*, and *Mig* states of buntings (*p < 0.05, Tukey’s post-test).

The experiment was conducted using male red-headed bunting (*Emberiza bruniceps*), as per the Committee for the Purpose of Control and Supervision of Experiments on Animals (CPCSEAs) guidelines and duly approved by the Chaudhary Charan Singh University, Institutional Animal Ethics Committee (IAEC Project Codes: IAEC/2020/02 and IAEC/2022/09). Red-headed buntings are night migratory songbirds which, over winter in the Indian subcontinent 28^o^N, exhibit a non-migratory diurnal state (*nMig*). With increasing day lengths in January to March, they overeat to go through a pre-migratory preparation state (*pMig*) before undertaking spring migratory (*Mig*) night flight to return to their breeding grounds 40^o^N. To begin with, acclimatized buntings (*n* = 18) were brought indoors and placed under a short photoperiod of 8 h of light and 16 h of dark (SD, 8L:16D) for 14 days in positive-pressure air-conditioned units (22°C ± 2°C, 50% relative humidity; monitored using Easy Log USB, Lascar electronics Inc. PA, United States) in three groups. Birds of group 1 (*n* = 6) were singly housed in well-ventilated activity recording cages, installed with passive infra-red sensor connected to the Chronobiology Kit hardware + software program system from Stanford Software Systems, Santa Cruz, CA, United States, that supports collection, plotting, and analyzing each bird’s flight behavior. Birds of group 2 and 3 were placed in groups (*n* = 6, each). Food (foxtail millet, *Setaria italica*, and egg mixture) and water were provided *ad libitum*, without direct animal handling. After 2 weeks of SD, birds of groups 1 and 2 were transferred to long days (LD, 14L:10D), while group 3 was retained under SD, as controls, whose body weight and relative mRNA gene expression level was simultaneously monitored (see Figures 1A, 1A.1–A1.3). The day of LD transfer was treated as day 0. For group 1, activity was continuously monitored and plotted as described earlier (Das and Gupta, 2016; Figures 1C, D). For group 2, body weight and blood sample collection was initiated on days 0, 7, and 28 when birds exhibited simulated non-migratory (*nMig*), pre-migratory (*pMig*), and migratory (*Mig*) annual life history states. About 25–50 μL of blood was drawn from the left brachial wing vein (see Gupta et al., 2020 for more details) and suspended in tubes with equal amounts of PBS (phosphate buffer saline) containing 5 mM EDTA. Nearly 32–36 h after the first blood collection, in group 2, 50 μL of blood was drawn from the right brachial wing vein for hematocrit analysis.

### Chemicals and other supplies

The anti CD71-PE, Annexin V-FITC, was purchased from Biolegend (San Diego, CA, United States). 5 (and 6) chloromethyl-2, 7-dichloro-dihydro-fluorescein diacetate (CM-H2DCFDA) and MitoTracker red (MTR) stain were procured from Molecular Probes, and Invitrogen (Eugene, OR, United States). All primers were commercially synthesized from Eurofins. Fetal bovine serum (FBS) was from Hyclone (South Logan, UT). For gene assay, PowerUp SYBR green, PCR Master Mix (Applied Biosystem, CA, United States), and RevertAid First Strand cDNA Synthesis Kit (Thermo Scientific™) were used. Analytical reagents such as RPMI and HEPES reagent were procured from Sigma-Aldrich (India). TRIzol reagent was procured from Ambion. All other chemicals were of analytical grade.

### Hematological analysis

Hemoglobin, PCV, and RBC (red blood cells) counts were assessed using standard procedures of spectrophotometry and calibration on Siemens ADVIA 2120 hematological analyzer and Sedgewick Rafter cells in a hemocytometer, respectively.

### Flow cytometric analysis

#### Estimation of cell size, intracellular ROS, and mitochondrial membrane potential

Blood was collected in PBS containing 5 mM EDTA and washed three times with ice-cold saline containing HEPES buffer (10 mM, pH-7.4) and 1% FBS. Erythrocyte area and width were measured by analyzing forward scattering A (FSC-A) and width (FSC-W) using flow cytometry. ROS levels were analyzed by staining the erythrocytes with the CM-H2DCFDA stain. In brief, erythrocytes were suspended in pre-warmed PBS + 2% FBS and incubated with CM-H2DCFDA stain (5 μM). The fluorescent product of CM-H2DCFDA was analyzed immediately by flow cytometry (Bhardwaj and Saxena, 2014; 2015; Goodchild and DuRant, 2020; Rajaura et al., 2022). MMP was measured by staining with 200 nM Mitotracker Red (MTR), followed by flow cytometric analysis (Montgomery et al., 2012; Bhardwaj and Singh, 2018).

#### Analysis of reticulocytes and apoptotic cells

Reticulocyte proportion was estimated by staining the cells with anti-transferrin (CD71) monoclonal antibodies, followed by flow cytometric analysis (Schmidt et al., 1986; Bhardwaj and Saxena, 2013). For apoptotic cell analysis, erythrocytes were resuspended in PBS containing 2.5 mM calcium chloride and stained with Annexin V-FITC antibody for 20 min at room temperature (Graham et al., 2015; Bhardwaj et al., 2022). Cells were washed and resuspended in PBS with 2.5 mM calcium chloride (CaCl_2_). After incubation (in the dark), cells were washed and analyzed using a FACSVerse flow cytometer and analyzed using Facsuite software. A minimum of 10,000 events (an event is the count of one cell) were analyzed using flow cytometry.

### Relative expression of metabolic genes in blood

#### RNA isolation and preparation of cDNA

Total RNA was extracted from blood using TRIzol reagent (Ambion) as per the manufacturer’s protocol. According to the TRIzol chloroform method, about 1 × 10^6^ erythrocyte cells were used for RNA isolation. The aqueous state containing RNA was separated and then precipitated by isopropyl alcohol. The RNA pellet obtained at the bottom of the centrifuge tube was washed twice with 75% ethanol, followed by air drying at room temperature. Nuclease-free water was used for suspending the RNA pellet and its purity was estimated by studying the absorbance of the obtained samples at 260/280 and 260/230 nm wavelengths in a Nanodrop spectrophotometer. The integrity of the isolated RNA was checked by running 5 µg of RNA on 1.2% formaldehyde agarose gel. After assessing its purity and integrity, RNA was further used for cDNA synthesis. For RT-PCR, cDNA was synthesized by using 1 µg of RNA per reaction mixture. A total of 1 µg RNA was treated with RNase-free DNase (Promega M6101) and reverse transcribed to synthesize cDNA using a cDNA synthesis kit (Thermo Scientific, K1622). cDNA integrity was verified using beta actin amplification followed by agarose gel electrophoresis; a sharp band of 750bp confirmed cDNA integrity.

#### Specific gene expression using qPCR

The amplification of cDNA was carried out by RT-qPCR. A total of 7 genes related with the fatty acid metabolism (ATGL, DGAT2, and GOT2), transporter (CD36 and FABP3), and ROS metabolism (SOD1 and NOS2) were investigated in the present study. mRNA levels were measured by quantitative PCR (RT-qPCR) using Applied Biosystems QuantStudio3 and SYBR green chemistry as described earlier (Singh et al., 2013; Trivedi et al., 2014). Briefly, a total reaction volume of 10 µL contained 1 µL each of cDNA (10 ng/μL) and forward and reverse primers (100 nm), 5 µL of PowerUp SYBR Green master mix, and 2 µL nuclease free water. β-actin mRNA expressions served as reference control to calculate and present relative mRNA expression levels (2^−ΔΔCT^, Livak & Schmittgen, 2001).

### Statistical analysis

Statistical data analysis was performed using GraphPad Prism software (Version 9). Data are shown as mean ± SEM (standard error of mean). Buntings are small birds, so values not detected due to less blood quantity were not included in analysis. Statistical significance was determined by two-way repeated measure ANOVA, followed by Tukey’s post-analysis, for state-wise comparison or comparing *pMig* and *Mig* with *nMig*, respectively. A *p* < 0.05 was considered significant.

## Results

All birds exhibited day activity, i.e., diurnal behavior, when placed under SD.

### Behavioral and physiological differences between *nMig*, *pMig*, and *Mig* buntings

Diurnal behavior continued for up to 15 days after transfer to LD in buntings, following which birds exhibited a significant increase in body weight (two-way repeated measure ANOVA, followed by Tukey’s post-hoc test revealed a difference, i.e., F_(2, 10)_ = 18.28; *p* < 0.001) (Figure 1B) and intense night activity (replication of *Zugunruhe* migratory activity in wild) (Figures 1C, D).

### Erythrocyte area increases during simulated migration

**FIGURE 2:**
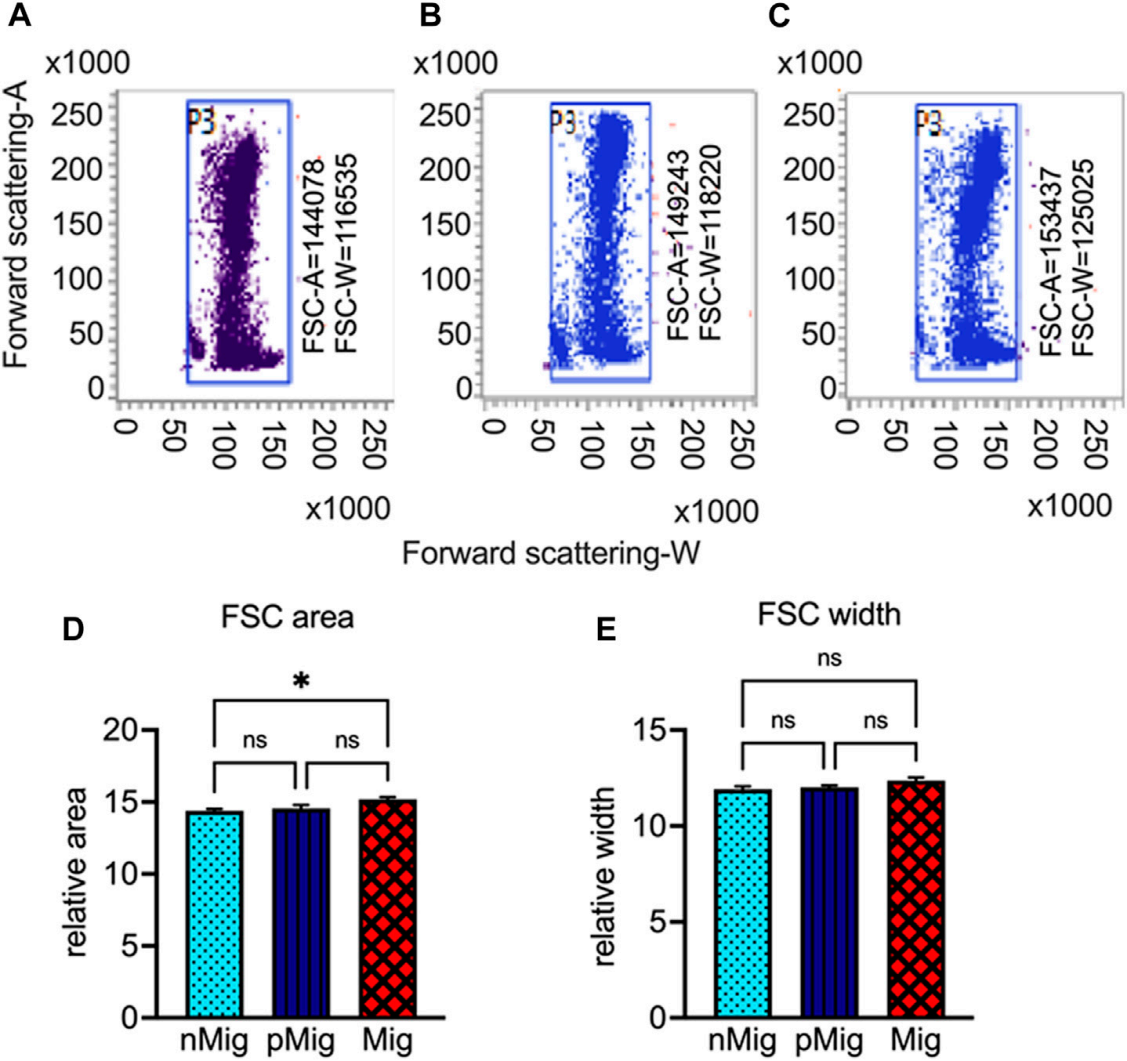
Changes in erythrocyte area and width during migration. Blood was collected from non-migratory (*nMig*, cyan blue), pre-migratory (*pMig*, blue), and migratory (*Mig*, red) red-headed buntings (*n* = 6) placed under SD/LD for 0, 7, and 28 days, and the mean area (forward scattering A) and width (forward scattering W) of erythrocytes were measured using flow cytometry. The dot-blots in Panel **(A–C)** show the FSC-A/FSC-W in non-migratory (*nMig*, A), pre-migratory (*pMig*, B), and migratory (*Mig*, C) states, respectively. The bar graphs in panels **(D,E)** depict the cumulative changes in erythrocyte area and width, respectively. The values in dot-plots indicate the mean FSC-A and FSC-W at different states. Data in **(D,E)** are represented as mean ± SEM. Asterisk (*) over the line on the bar indicates a significant difference between *nMig*, *pMig*, and *Mig* states of buntings (*p < 0.05, Tukey’s post-test).

A significant increase in hematocrit, i.e., hemoglobin (F_(2, 10)_ = 23.23; *p* < 0.0005, Figure 1E), packed cell volume (F_(2, 10)_ = 7.003; *p* < 0.05, Figure 1F), and RBC count (F_(2, 10)_ = 7.064; *p* < 0.05, Figure 1G), was observed during simulated migration. Also, the flow cytometric data showed enhanced erythrocyte area, but not width (Figures 2A–E), i.e., the mean erythrocyte area was 144,078 in *nMig* (Figures 2A, D), which increased to 149,243 in *pMig* (Figures 2B, D), with further enhancing to 153,437 during *Mig* (Figures 2C, D), showing 5.5% increase in the erythrocyte area. The erythrocyte width did not increase with change from *nMig* to *Mig* (Figure 2E).

### The reticulocyte production and reactive oxygen species level increased during simulated migration

**FIGURE 3:**
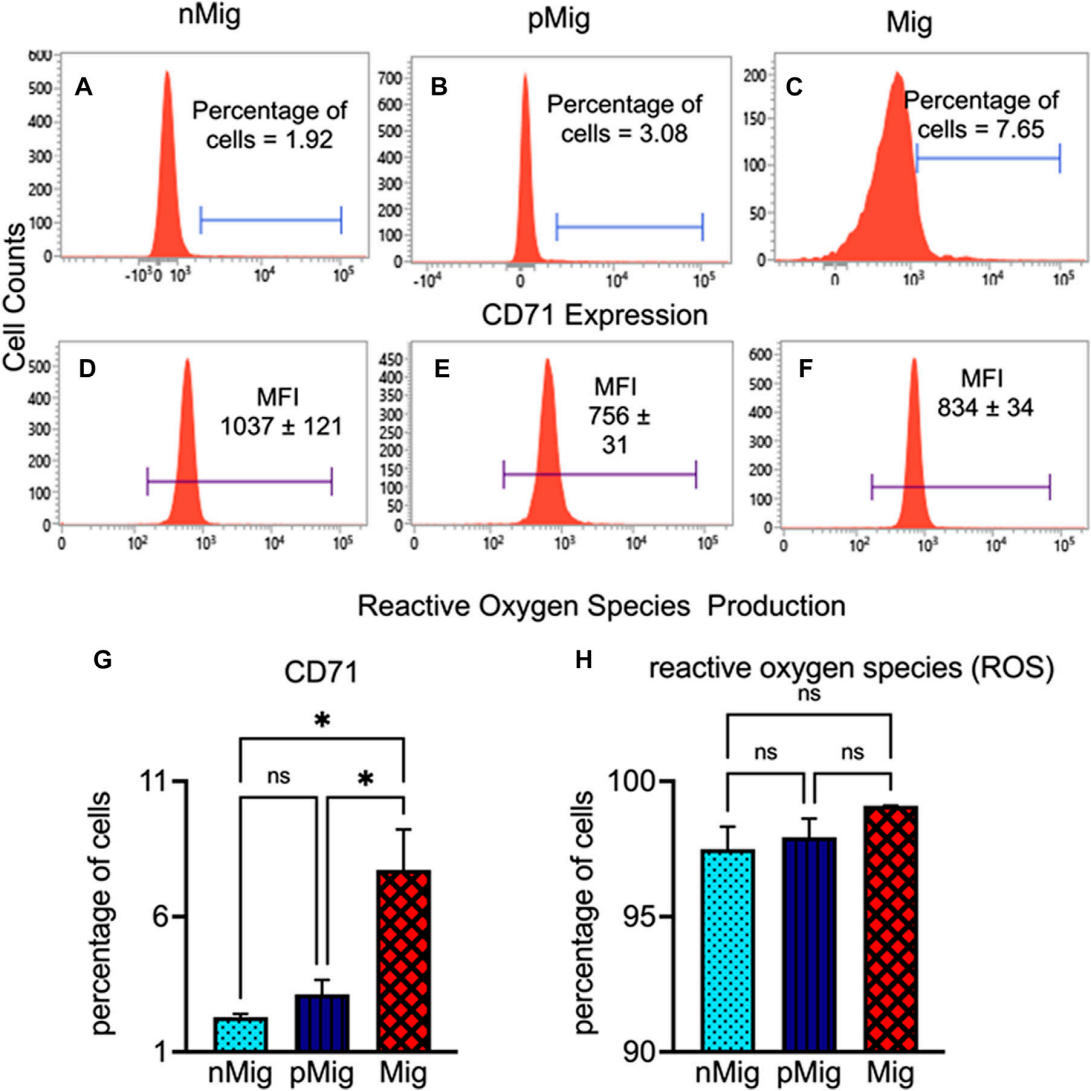
Modulation of reticulocyte production and ROS accumulation in peripheral blood circulation. The blood was isolated from non-migratory (*nMig*, cyan blue), pre-migratory (*pMig*, blue), and migratory (*Mig*, red) red-headed buntings (*n* = 6) placed under SD/LD for 0, 7, and 28 days. Newly formed reticulocytes were estimated by staining with anti-CD71-PE monoclonal antibody, and ROS accumulation was measured by staining with the CM-H2DCFDA stain. The flow cytometric histograms in Panel **(A–C)** show the reticulocyte proportion in *nMig*, *pMig* and *Mig* states, respectively. The ROS level in corresponding states has been depicted in panels **(D–F)**. The bar graph in panels **(G,H)** shows the cumulative alteration in the reticulocyte and ROS production. The horizontal blue line in panel **(A–C)** represents CD71-positive cells. In panels **(D–F)**, the blue lines correspond to the mean fluorescence intensity (MFI) of CM-H2DCFA stain. Data are represented as mean ± SEM. Asterisk (*) over the line on the bar indicates a significant difference between *nMig*, *pMig*, and *Mig* states of buntings (*p < 0.05, Tukey’s post-test).

The simulated migration in bunting causes the increased production of reticulocytes in the blood. The proportions of reticulocytes (CD71^+ve^) were 1.92% in *nMig* birds, which increased to 3.08% in the *pMig* state (Figures 3A, B). As the bird switches from *pMig* to *Mig* state, the reticulocyte production further increased more than two-fold as compared to the *pMig* state (Figures 3B, C). The cumulative data show a 4.8-fold enrichment of reticulocytes in between *nMig* and *Mig* transition (Figure 3G) (two-way repeated measure ANOVA, followed by Tukey’s post-hoc test revealed significant difference, i.e., F _(2, 8)_ = 8.47; *p* < 0.05)*.*

In comparison to reticulocyte production, the ROS-positive cell percentages did not change significantly between *nMig*, *pMig*, and *Mig* (Figure 3H)*.* However, the mean fluorescence intensity (MFI) showed a significant decline in the ROS production from *nMig* to *pMig* state transition. The ROS level was maximum in *nMig* groups, which decreased in *pMig* and *Mig* states. The flow cytometric histogram represents the MFI of ROS production in *nMig* was 1,037, which declined to 756 and 834 in the *pMig* and *Mig* groups, respectively (Figures 3D–F).

### MMP and apoptosis in erythrocytes during simulated migration

**FIGURE 4:**
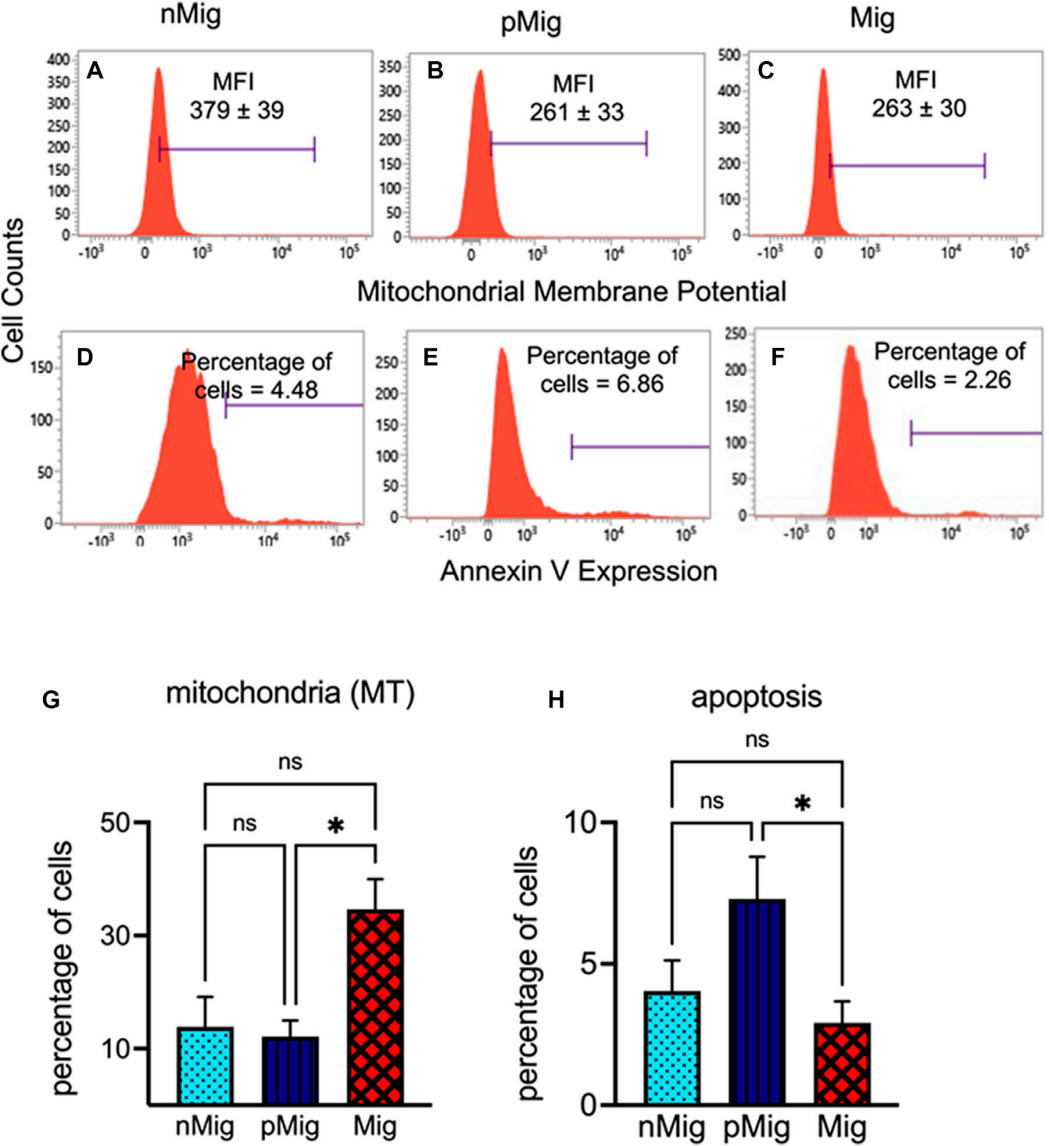
Alteration in the mitochondrial membrane potential (MMP) and apoptosis in erythrocytes in circulation. The blood was collected from non-migratory (*nMig*, cyan blue), pre-migratory (*pMig*, blue), and migratory (*Mig*, red) red-headed buntings (*n* = 6) placed under SD/LD for 0, 7, and 28 days. MMP erythrocytes were determined by staining with MitoTracker red dye. Apoptotic cells were quantified using Annexin V staining followed by flow cytometric analysis. Flow cytometric histograms in panels **(A–C)** show the MMP in *nMig*, *pMig*, and *Mig* states, respectively. The proportion of apoptotic cells in corresponding states has been depicted in panels **(D–F)**. The bar graph in panels **(G,H)** shows the cumulative changes in mitochondrial membrane potential and apoptotic cell proportion. The horizontal blue line in panel **(A–C)** represents the mean fluorescence intensity (MFI) of mitotracker stain. In panels **(D–F)**, it corresponds to the percentages of Annexin V-positive cells. Data are represented as mean ± SEM. Asterisk (*) over the line on the bar indicates a significant difference between *nMig*, *pMig*, and *Mig* states of buntings (*p < 0.05, Tukey’s post-test).

MMP decreased as the bird switched from *nMig* to *pMig* and *Mig* states (Figures 4A–C). Histograms in Figure 4 show that the MFI of Mitotracker stain in *nMig* erythrocytes was 379, which declined to 261 in the *pMig* state. Furthermore, as birds switched to simulated migration, the MFI level was almost similar to the *pMig* state. However, a significant increase in Mitotracker (MT)-positive cells proportion was seen during the *pMig* to *Mig* state transition but not in *nMig* to *pMig* transition (Figure 4G) (two-way repeated measure ANOVA, followed by Tukey’s post-hoc test revealed significant difference, i.e*.*, F_(2, 8)_ = 5.62; *p* < 0.05, between *nMig, pMig*, and *Mig*)*.*

Changes in ROS and MMP may lead to the apoptosis of cells in the circulation. We calculated the apoptosis by staining the cells with Annexin V. The proportion of apoptotic cells reduced during the *Mig* state was 6.86%–2.26%) (Figures 4D–H). Two-way repeated measure ANOVA followed by Tukey’s post-hoc test revealed significant (F_(2, 10)_ = 6.06; *p* < 0.05) difference between *nMig*, *pMig*, and *Mig* states*.* There was 62% decline in apoptotic cell % in between *pMig* and *Mig* states.

### Changes in the relative expression of metabolic genes in blood

**FIGURE 5:**
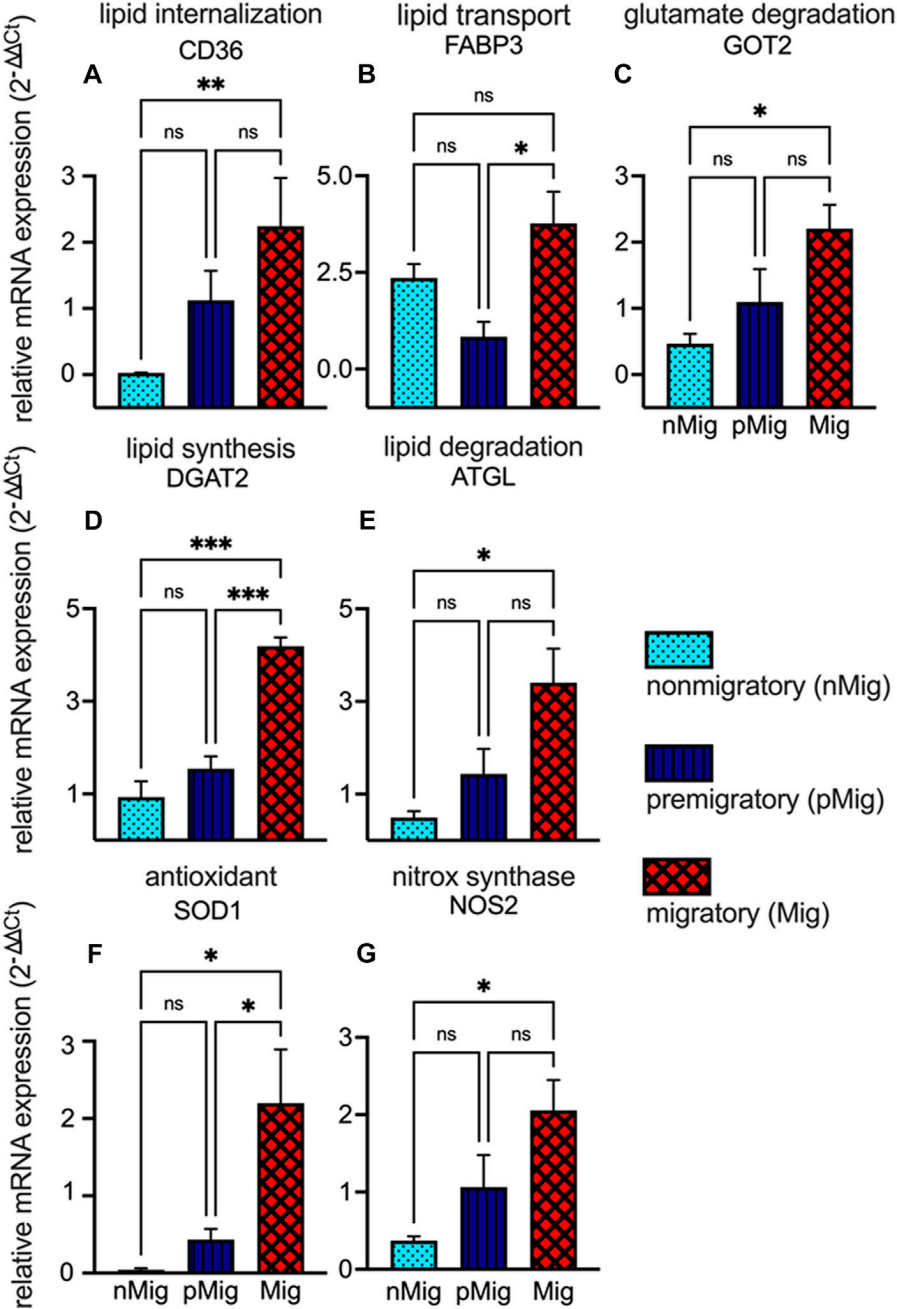
Changes in the relative expression of fatty acid transporters and antioxidant genes. The blood was collected from non-migratory (*nMig*, cyan blue), pre-migratory (*pMig*, blue), and migratory (*Mig*, red) red-headed buntings (*n* = 6) placed under SD/LD for 0, 7, and 28 days. RNA was isolated from 8L:16D/14L:10D exposed birds erythrocytes using TRIzol (TRI) reagents. The first strand of cDNA for isolated RNA was synthesized by using the First Strand cDNA Synthesis kit. The cDNA obtained was amplified using qRT-PCR. The RNA transcripts for different genes were quantified by SYBR Green method. Relative expression levels of fatty acid transporter genes CD36, FABP3, ATGL, DGAT2, and GOT2 have been shown in panels **(A–E)**. The expression levels of antioxidant genes SOD1 and NOS2 are shown in panels **(F,G)**. Expression levels of mRNA were normalized to β-actin levels using the 2^−ΔΔCT^ method. Data are represented as mean ± SEM. Asterisk (*) over the line on the bar indicates a significant difference between *nMig*, *pMig*, and *Mig* states of buntings (*p < 0.05, Tukey’s post-test).

The expression levels of antioxidant enzymes superoxide dismutase (SOD1), nitric oxide synthase (NOS2), fatty acid transporter (CD36), fatty acid binding protein (FABP3), adipose triglyceride lipase (ATGL), diacylglycerol acyl transferase (DGAT2), and glutamic-oxaloacetic transaminase (GOT2) were studied in different states. The mRNA expression levels of CD36 significantly increased (F_(2, 10)_ = 9.06; *p* < 0.005, two-way repeated measure ANOVA, followed by Tukey’s post-hoc test) from *nMig* to *Mig* states (Figure 5A). FABP3 mRNA expression was highest in the *Mig* state. Two-way repeated measure ANOVA followed by Tukey’s post-hoc test revealed a significant (F_(2,6)_ = 6.92; *p* < 0.05) difference between *pMig* and *Mig* states with a 4.4-fold increase in the latter state (Figure 5B). GOT2 mRNA expression increased from *nMig* to *Mig* states. Two-way repeated measure ANOVA followed by Tukey’s post-hoc test revealed significant (F_(2,7)_ = 8.98; *p* < 0.05) difference in GOT2 expression levels between *nMig* and *Mig* states with a 4.7-fold increase in the latter state (Figure 5C). DGAT2 mRNA expression increased from *nMig* to *Mig* states. Two-way repeated measure ANOVA followed by Tukey’s post-Hoc test revealed a significant (F_(2,4)_ = 118.4; *p* < 0.0005) difference between *nMig* and *Mig* states with 4.4-fold increase in the latter state (Figure 5D). ATGL mRNA expression increased from *nMig* to *Mig* state. Two-way repeated measure ANOVA followed by Tukey’s post-hoc test revealed a significant (F_(2, 7)_ = 5.306; *p* < 0.05) difference between *nMig* and *Mig* states with a 6.5-fold increase in the latter state (Figure 5E). SOD1 mRNA expression shows increase from *nMig* to *Mig* states. Two-way repeated measure ANOVA followed by Tukey’s post-hoc test revealed significant (F_(2, 8)_ = 7.668; *p* < 0.05) change in SOD1 expression levels between *nMig* and *Mig* states with 31-fold change (Figure 5F). Changes in NOS2 expression were similar to those of SOD1. It was least in the *nMig* state and increased during transition to the *Mig* state. Two-way repeated measure ANOVA followed by Tukey’s post-hoc test revealed significant difference between *nMig* and *Mig* states (F_(2, 5)_ = 7.751; *p* < 0.05) with a 5.5-fold change (Figure 5G).

## Discussion

Erythrocytes are the major transporter of O_2_ from the heart to various organs. They play a vital role in energy metabolism (Malkoc et al., 2021) in migratory birds. Migration is an energetically costly behavior that drives need-based erythrocyte modulation to deliver oxygen (Soulsbury et al., 2021). Molecular underpinnings of erythrocyte-associated cellular dynamics were assessed in night migratory buntings during different physiological states of spring migration, wherein physiological and behavioral changes of body weight and daily activity profiles conform to those reported in earlier studies.

Hematocrit and forward scattering area (FSC-A) and width (FSC-W) of erythrocytes in *nMig*, *pMig*, and *Mig* states corresponded to the energy requirements such that a significant increase in area during transition from *nMig* to *Mig* states occurred. Reasonably, increased cell area can accommodate more hemoglobin to enrich oxygen and CO_2_ transport. Soulsbury et al. (2021) reviewed 631 bird species’ data to conclude elongated erythrocytes during migration. Our estimation of CD71^+Ve^ erythrocytes (reticulocytes) in different states of simulated migration highlights naive erythrocytes (reticulocytes) that freshly enter the circulation. The reticulocytes increased significantly from *nMig* to *Mig* states. The enhanced reticulocyte production might be a compensatory response to the metabolic demand and attrition due to oxidative stress (Jenni-Eiermann et al., 2014). We speculated that by the time buntings enter full-fledged migration, they adapt physiologically to recover from the erythrocyte deficiency through erythrocyte regeneration.

The relative level of ROS production in erythrocytes in *nMig*, *pMig*, and *Mig* states of simulated migration, despite extreme oxidative stress, did not vary as hypothesized. Our data suggest that though the ROS-positive cell percentages exhibited minor variations, the MFI of ROS significantly reduced in *pMig* and *Mig* states, compared to the *nMig*. In mammals, the erythrocyte production switches from bone marrow to spleen, facilitating resistance to oxidative stress (Bhardwaj and Saxena, 2014; 2015). Herein, a low ROS level in erythrocyte could be a strategy to combat oxidative stress during simulated migration, but this needs substantiation through further investigation.

Mitochondria are dually engaged in energy budgeting and ROS production. The proportion of MT-positive cells percentage increased significantly from *pMig* to *Mig* states. Increased energy demand justifies the increase in the percentage of MT cells. The MFI of MT, just like ROS MFI, declined in *pMig* birds, remaining almost similar until *Mig* transition. Erythrocyte ROS MFI aligned with MT MFI with simultaneous overexpression of SOD1 mRNA levels during migration (Dimayuga et al., 2007), suggesting intracellular redox signaling modulated the mitochondrial membrane potential.

The decline in MMP is also associated with increased mortality (Jeong and Seol, 2008; Wang and Youle, 2009). Studies relating mitochondrial activity to apoptosis in erythrocytes are limited to heat stress investigations in chickens, wherein higher temperatures caused morphological alterations and increased the activity of pro-apoptotic caspases in erythrocytes (Szabelak et al., 2021). Our investigation of erythrocyte apoptosis using Annexin V-positive cell percentages suggested altered apoptosis until migratory preparation, which stabilized during simulated migratory state, again suggesting birds’ ability to maintain erythrocytes survival at the basal level.

Furthermore, we have also analyzed the molecular changes in ROS-related and fatty acid gene expression in various states. Erythrocytes contain a pool of antioxidant enzymes that scavenge free radicals. We observed a rise in erythrocyte SOD1 mRNA levels during simulated migration as also suggested in hypothalamus of buntings during migration (Sharma et al., 2021). Nitric oxide synthase (NOS2) is a ROS indicator. Nitric oxide (NO) is a small free radical with critical signaling roles in regulation of healthy vasculature performance, which, in humans, is supplemented by nitrite reduction pathways under hypoxia (Tejero et al., 2019). These suggestions improved the ability of bird vasculature to combat exercise stress. In birds also, NOS2 increase indicates increased proteolysis, which might be related to increased energy demands.

The expression of fat metabolism-related genes was also modulated in erythrocytes. The integral membrane glycoprotein CD36 is a fatty acid translocase (FAT), which plays an important role in the transportation of fatty acids (FAs) for energy production (Ibrahimi et al., 1999; Ibrahimi and Abumrad, 2002). CD36 mRNA expression increased from *nMig* to *Mig*. The increase in CD36 expression in erythrocytes might be due to energy accumulation, carrying fat droplets to adipose tissue (Araújo et al., 2022) as *Mig* preparation, which results from continuous pre-migratory hyperphagia (Jain and Kumar, 1995).

The fatty acid binding protein (FABP3) exhibits tissue-specific aspects of lipid and fatty acid metabolism. FABP3 is a fatty acid carrier in blood, whose mRNA expression began to alter during *pMig* but enhanced significantly from *pMig* to *Mig* states. The upregulation of FABP3 expression is correlated with the increased utilization of FA during migration (Gugleilmo, 2010; Gupta et al., 2020). The mRNA expression of ATGL, DGAT2, and GOT2 also increased from *nMig* to *Mig* transition with much overexpression during *Mig.* The increased ATGL expression indicated enhanced turnover of fatty acids. GOT2, a pyridoxal phosphate-dependent enzyme participating in the malate aspartate shuttle of electron transport chain, indicated more aerobic metabolism to produce energy for migration. The increased DGAT2 expression indicated promotion of lipid internalization. To sum up, an increase in erythrocyte area, increased reticulocyte production, and adaptive changes in ROS and MMP occurred in erythrocytes during simulated migration. To support the metabolic efficiency of erythrocytes in avian migrants, the molecular data suggested increased expression of antioxidant and fat metabolizing genes *via nMig* to *Mig* transition.

Conclusion: taken together, nucleated and mitochondria-containing erythrocytes of red-headed buntings exhibit metabolic ability for enhanced energy metabolism and support ROS and apoptosis reduction by maintaining the 1) erythrocyte threshold through erythropoiesis, 2) adaptively reducing ROS consequences, and 3) modulating antioxidant function to support mitochondrial hyperactivity. This is the first study involving the study of erythrocyte ROS dynamics alongside molecular underpinnings of metabolic regulation in the blood of a long-distance obligate avian migrant.

## Data Availability

The raw data supporting the conclusion of this article will be made available by the authors, without undue reservation.
