# Supplementary figures and images for: Altered dynamics of mitochondria and reactive oxygen species in the erythrocytes of migrating red-headed buntings
Source: Front Physiol. 2023 Feb 9;14:1111490. doi: 10.3389/fphys.2023.1111490 (PMC9947641; doi:10.3389/fphys.2023.1111490)

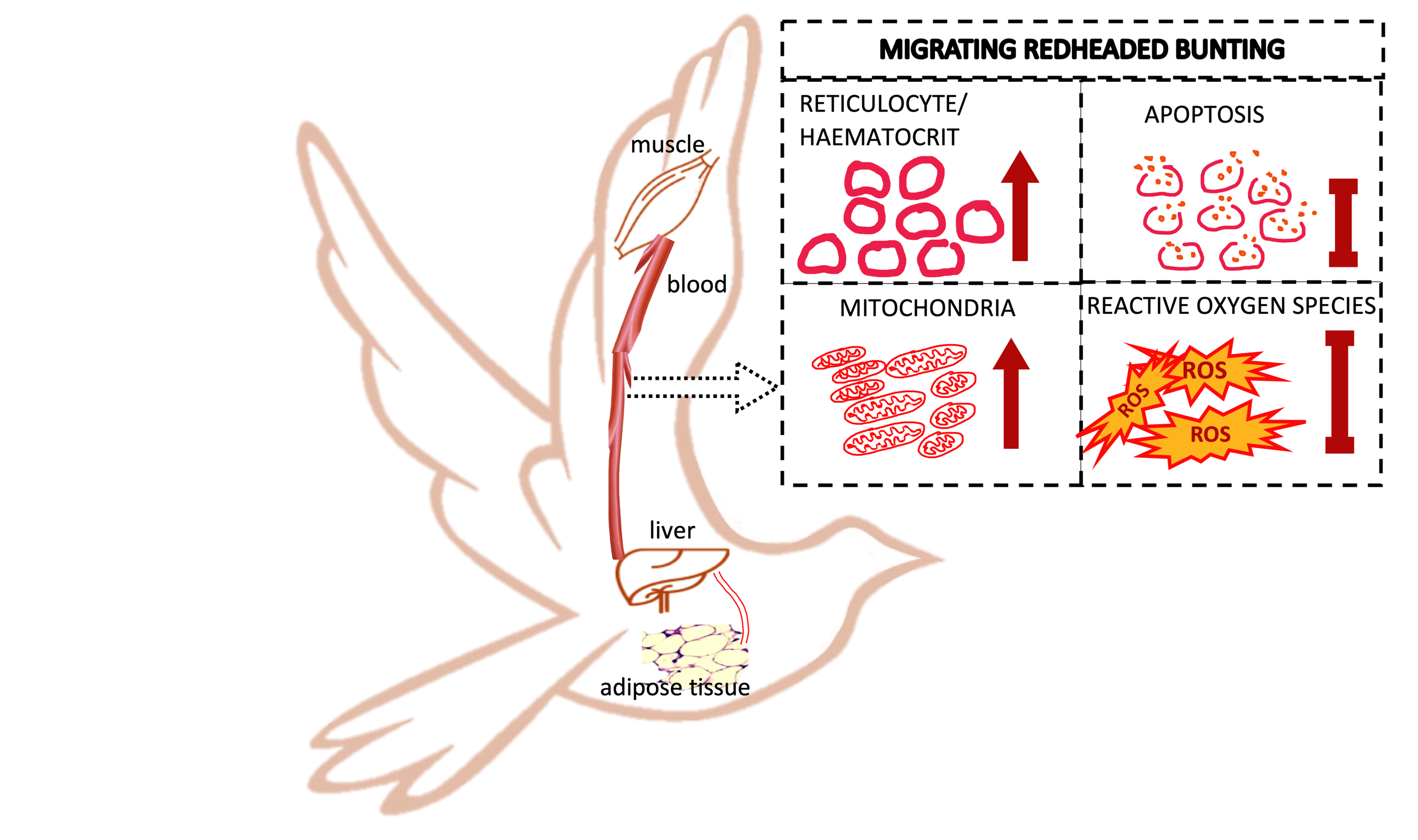

Supplement: Supplementary file 1 [file Image1.TIFF]
